# Association of type 2 diabetes with anthropometrics, bone mineral density, and body composition in a large-scale screening study of Korean adults

**DOI:** 10.1371/journal.pone.0220077

**Published:** 2019-07-24

**Authors:** Jeong Hee Chi, Moon Sun Shin, Bum Ju Lee

**Affiliations:** 1 Department of Software, Konkuk University, Seoul, Republic of Korea; 2 Department of Computer Engineering, Konkuk University, Chungju, Republic of Korea; 3 Future Medicine Division, Korea Institute of Oriental Medicine, Deajeon, Republic of Korea; University of Extremadura, SPAIN

## Abstract

**Objectives:**

Type 2 diabetes mellitus (T2DM) is a common, chronic disease that is closely associated with anthropometric indices related to obesity. However, no study published to date has simultaneously examined the associations of T2DM with anthropometrics, bone mineral density (BMD), and body composition variables. The present study aimed to evaluate the associations of T2DM with anthropometrics, BMD and body composition variables and to identify the best indicator of T2DM in Korean adults.

**Methods:**

The data used in this study were obtained from the Korea National Health and Nutrition Examination Survey conducted from 2008 to 2011. A total of 7,835 participants aged from 40 to 90 years were included in this study. A binary logistic regression analysis was performed to examine the significance of differences between the groups with and without T2DM, and the areas under the receiver operating characteristic (AUCs) curves were calculated to compare the predictive power of all variables.

**Results:**

In men, waist-to-height ratio (WHtR) displayed the strongest association with T2DM (adjusted odds ratio (OR) = 1.838 [1.513–2.233], adjusted p<0.001), and waist circumference (WC) and WHtR were the best indicators (WC: AUC = 0.662 [0.639–0.685], WHtR: AUC = 0.680 [0.658–0.703]) of T2DM among all the variables. In women, left leg (LL) and right leg (RL) fat displayed strong negative associations with T2DM (LL fat: adjusted OR = 0.367 [0.321–0.419], adjusted p<0.001, RL fat: adjusted OR = 0.375 [0.329–0.428], adjusted p<0.001), and WC and WHtR were excellent indicators (WC: AUC = 0.730 [0.709–0.750], WHtR: AUC = 0.747 [0.728–0.766]) of T2DM among all the variables. In particular, the WHtR in men and LL and RL fat in women exhibited the strongest associations with T2DM, and the predictive power of the WC and WHtR was stronger than BMD, fat, and muscle mass variables in both men and women. Additionally, the predictive power of the WC and WHtR was stronger in women than in men.

**Discussion:**

Of the anthropometric indices, BMD, and body fat and muscle variables, the best indicators of T2DM were WC and WHtR in both Korean men and women. The results of the present investigation will provide basic information for clinical studies of patients with T2DM and evidence for the prevention and management of T2DM.

## Introduction

Diabetes is a common, chronic disease worldwide, and the number of patients continues to increase due to reduced physical activity and increased obesity [1]. The World Health Organization (WHO) has reported that the number of adults with diabetes in the world has increased from 108 million in 1980 to 422 million in 2014 [2]. The Korea Diabetes Association has reported that the prevalence of diabetes (fasting plasma glucose≥126 mg/dL, HbAlC≥6.5%) in Korean adults aged ≥30 years increased from 12.4% in 2011 to 14.4% in 2016 and that the diabetic population is expected to reach approximately 6 million in 2050 [3]. In particular, the prevalence of diabetes mellitus is more than 10% in men aged ≥40 years and women aged ≥50 years. It is important to maintain proper blood glucose control in diabetes because it is likely to cause various complications such as chronic renal failure, vision loss, hypertension, dyslipidemia, heart failure, myocardial infarction, ischemic heart disease and stroke [3–6]. In this study, we focus on type 2 diabetes (T2DM) rather than type 1 diabetes mellitus (T1DM) or and gestational diabetes. T2DM is a disease that affects 90–95% of all adult diabetes patients and is caused by a lack of insulin secretion from the β-cells of the pancreas or by insulin resistance that arises from the inability of insulin to act normally in regulating nutrient metabolism in peripheral tissues [5]. Although the prevalence of diabetes continues to increase, there are still many patients who do not know they have the disease until complications arise. According to the International Diabetes Federation in 2017, an estimated 50% of all people aged 20–79 years with diabetes are unaware of their disease [7], and the Korea Diabetes Association has reported that 3 out of 10 diabetic patients are unaware of their disease [3]. Therefore, it is essential to develop indicators that can identify diabetes as early as possible based on various data from participants.

Many studies to date have assessed the association of T2DM with anthropometric indices, bone mineral density (BMD), and body composition variables. Among the anthropometric indices, many studies have reported the strongest association between the waist-to-height ratio (WHtR) and T2DM [8–15]. According to several studies, waist circumference (WC) [16–18] and body mass index (BMI) [9,17] are strongly associated with T2DM. In addition, some studies have reported that neck circumference (NC) in Brazilian women [19] and the Chinese visceral adiposity index (CVAI) in Chinese adults are strongly associated with T2DM [20]. Age has the greatest impact on diabetes in Ghana [21]. A study has shown that prediction of the fasting plasma glucose status using a combination of anthropometric measures was superior to individual measures alone [22]. Regarding body composition variables, some studies have reported that the association of T2DM with central fat or abdominal fat is stronger than the association with general fat [23–26]. Leg fat strongly correlates with T2DM [27]. With respect to BMD parameters, several studies have reported that T2DM is associated with a higher BMD and, paradoxically, with increased fracture risk [28–36], while several studies have shown that diabetes affects bone loss [37,38]. In addition, some investigations have shown that patients with both T2DM and fractures have a low BMD [39,40], and several studies have reported that T2DM is not associated with a higher prevalence or incidence of vertebral fractures in older men [41,42]. Therefore, the association of T2DM with BMD remains inconsistent across studies. Additionally, although many studies have been conducted to find useful indicators of T2DM, the previous studies were based on only partial information, such as anthropometrics, BMD, and body fat mass. Comparisons of T2DM with various indicators are essential for finding the indicator with the greatest predictive power. Therefore, the aim of this study is to identify the associations and predictive power between T2DM and various indicators such as anthropometric indices, BMD, and body composition parameters. The results of the present investigation will provide basic information for clinical studies of patients with T2DM and evidence for the prevention and management of T2DM.

## Materials and methods

### Study population and data source

The data used in this study were obtained from the 2008–2011 Korea National Health and Nutrition Examination Survey (KNHANES IV-2, 3 and V-1, 2), which is a prospective, cross-sectional, nationally representative survey study conducted by the Korea Centers for Disease Control and Prevention [43,44]. The KNHANES datasets were approved by the Korea Ministry of Health and Welfare (2008-04EXP-01-C, 2009-01CON-03-2C, 2010-02CON-21-C, and 2011-02CON-06-C). The institutional review boards of the Korea Institute of Oriental Medicine and Konkuk University approved the access and analysis of open source data from the KNHANES in this study, with a waiver of documentation of informed consent (IRB No. I-1805/003-001 and 7001355-201802-E-063).

Health interviews and health examinations included in the KNHANES were performed at mobile examination centers (MECs). The health interviews, which requested information such as medical conditions, housing characteristics, socioeconomic status and alcohol use, were collected by a face-to-face interview and self-administered form. Health examinations requesting information such as height, weight, blood test and BMD were also measured at MECs [45].

**Fig 1:**
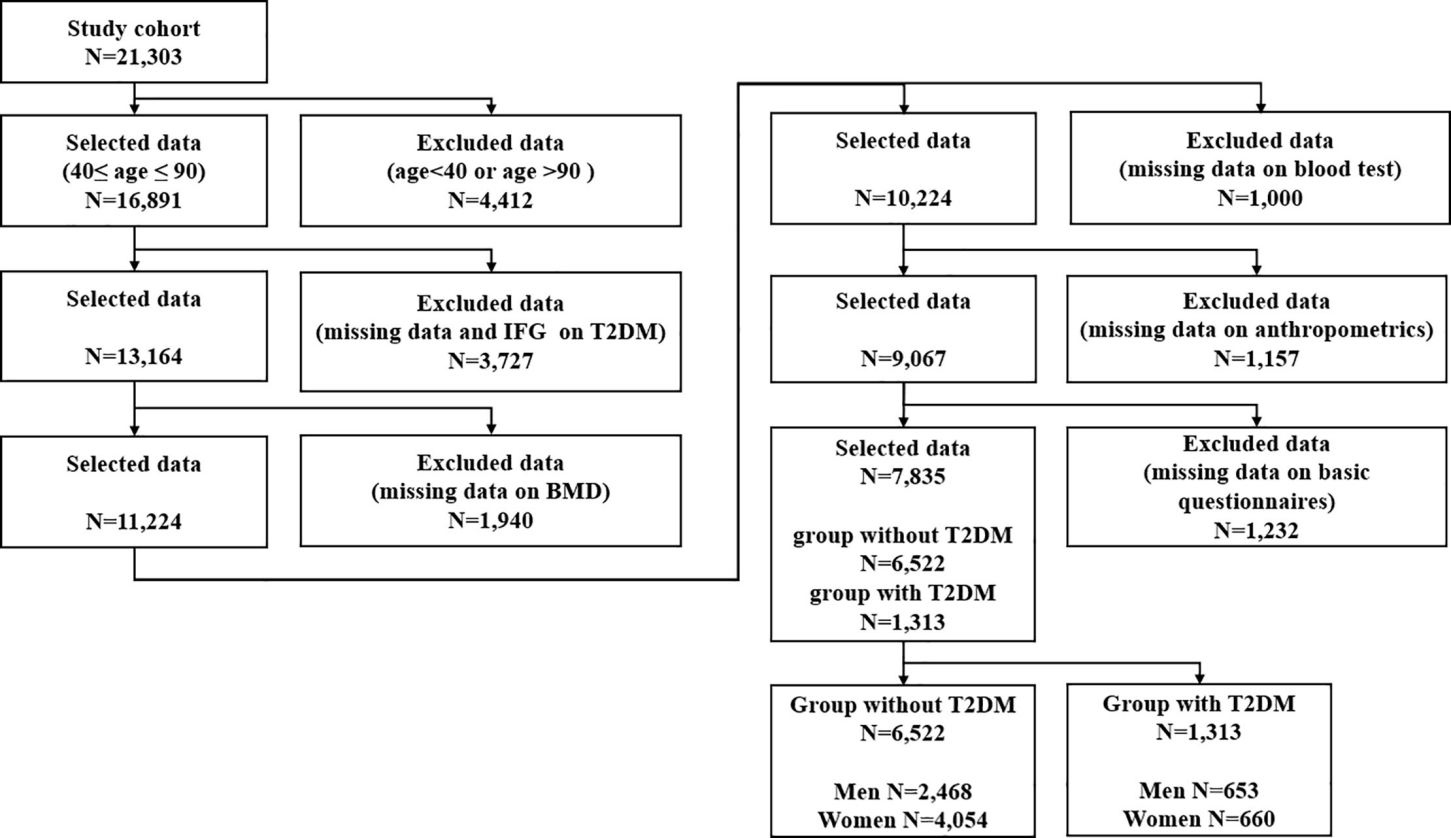
Sample selection procedure. T2DM, type 2 diabetes mellitus; IFG, impaired fasting glucose; BMD, bone mineral density.

The KNHANES I—VII were conducted from 1998 to 2016. BMD examinations were performed only from 2008 to 2011; therefore, we chose the KNHANES IV-2, 3 and V-1, 2 to find associations of T2DM with various bone and body composition variables. The KNHANES IV-2, 3 and V-1, 2 included 21,303 participants who completed blood tests, bone densitometry and body fat composition tests, along with a health examination. According to the Korean Diabetes Association [3], the prevalence of diabetes increases rapidly at age >40 years. T2DM is the most common type of diabetes, which affects 90–95% of all patients with diabetes [5,46]. Therefore, in the present study, we focused on participants aged 40 to 90 years with T2DM. The sample included 16,891 participants ranging in age from 40 to 90 years; 3,727 participants with impaired fasting glucose (IFG) levels, T1DM or missing values for T2DM were excluded. The KNHANES did not clearly distinguish T1DM from T2DM, and therefore, participants reporting a first diagnosis of diabetes at an age <30 years who started insulin therapy within 1 year of diagnosis were considered as having T1DM, in accordance with a previous study [47]. In total, 5,329 participants with missing values for BMD (1,940), blood tests (1,000), anthropometrics (1,157) and basic questionnaires (1,232) were excluded, and a sample of 7,835 participants was finally collected. The final sample consisted of 3,121 men (normal: 2,468 and T2DM: 653) and 4,714 women (normal: 4,054 and T2DM: 660). Fig 1 shows a detailed schematic of the data preprocessing procedure.

### Definition

Diabetes mellitus is defined as a metabolic disease characterized by hyperglycemia resulting from defects in insulin secretion, insulin action, or both [5]. WHO and previous studies have reported that diabetes mellitus is defined as a fasting plasma glucose≥126 mg/dL, 2-hour plasma glucose≥200 mg/dL, or HbA1C≥6.5% [3,5]. In this study, the diagnosis of T2DM was based on a fasting plasma glucose level≥126 mg/dL, HbA1C level≥6.5%, the use of antidiabetic medications or glucose-lowering drugs, or physician-diagnosed diabetes.

### Measurements

All anthropometric measurements, such as height, weight, WC, BMI, and WHtR, were recorded using standard methods. Height was measured to the nearest 0.1 cm using a Seca 225 portable stadiometer (Seca, Hamburg, Germany), and weight was measured with an accuracy of 0.1 kg using an electronic scale GL-6000-20 (Caskorea, Seoul, Korea). WC was measured at the midline between the lower rib margin and iliac chest to the nearest 0.1 cm. BMI was calculated as weight (kg)/square of height (m^2^). WHtR was calculated as WC divided by height. Blood samples were collected from the antecubital vein after an 8-hour fast. Fasting plasma glucose, total cholesterol, triglyceride (TG, ≥12 hour fasting), and high-density lipoprotein-cholesterol (HDL) levels in all participants were measured enzymatically using a Hitachi Automatic Analyzer 7600 (Hitachi, Tokyo, Japan). The visceral adiposity index (VAI), a gender-specific index based on WC, BMI, TG and HDL, was calculated as follows [48]:

$$men:VAI=\left(\frac{WC}{39.38+(1.88+BMI)}\right)\times \left(\frac{TG}{1.03}\right)\times \left(\frac{1.31}{HDL}\right)$$

$$women:VAI=\left(\frac{WC}{36.58+(1.89+BMI)}\right)\times \left(\frac{TG}{0.81}\right)\times \left(\frac{1.52}{HDL}\right)$$

BMDs of the total femur, trochanter, intertrochanter, femoral neck, ward, lumbar spine, and whole body were measured using dual energy X-ray absorptiometry (DISCOVERY QDR-4500W fan-beam densitometer, Hologic, Inc., Bedford, MA, USA). Body fat composition was measured using the same equipment and methods as used for BMD. Body fat mass, lean body mass, weight (mass) and body fat percentage were measured in the trunk, left leg (LL), right leg (RL) and whole body. The lean body mass is the fat-free mass including the bone mineral content. Muscle mass (g) was calculated as the lean body mass minus the bone mineral content, assuming that all non-fat and non-bone tissue is muscle.

### Statistical analysis

Statistical analyses were performed using SPSS 21 for Windows (SPSS, Inc., Chicago, IL, USA). In crude analyses and in the analyses adjusted for age and BMI, a binary logistic regression analysis was performed to identify associations between the group without T2DM and group with T2DM after applying standardized transformations to the datasets. Independent two-sample t-tests were performed to assess gender differences in statistical characteristics. Table 1 shows a detailed description of the demographic characteristics and values for all study variables in each group. The area under the receiver operating characteristic (AUC) curve is a major criterion for comparisons of the predictive ability of individual measures. Therefore, the present study used AUCs as indicators to evaluate whether a clinically meaningful improvement in the discrimination of T2DM was achieved by anthropometric, BMD, body fat and muscle variables.

**Table 1:** Demographic characteristics and values for all study variables.

Variables		Men		Women	
		group without T2DM	group with T2DM	group without T2DM	group with T2DM
Numbers		2468	653	4054	660
Age (mean, SD)		54.88 (11.65)	60.38 (10.22)	54.55 (11.29)	63.38 (10.32)
Residential areas (no. of participants, %)					
	City	1777 (72.00)	476.0 (72.90)	2968 (73.21)	469.0 (71.10)
	Rural	691.0 (28.00)	177.0 (27.10)	1086 (26.79)	191.0 (28.90)
Education (no. of participants, %)					
	< = Elementary school	567.0 (22.97)	177.0 (27.10)	1531 (37.77)	454.0 (68.80)
	Middle school	370.0 (14.99)	139.0 (21.30)	598.0 (14.75)	75.00 (11.40)
	High school	799.0 (32.37)	213.0 (32.60)	1282 (31.62)	107.0 (16.20)
	> = University	732.0 (29.66)	124.0 (19.00)	643.0 (15.86)	24.00 (3.600)
Occupation (no. of participants, %)					
	White-collar worker	362.0 (14.70)	62.00 (9.500)	281.0 (6.900)	7.000 (1.054)
	Office worker	237.0 (9.600)	35.00 (5.344)	145.0 (3.600)	10.00 (1.506)
	Service	304.0 (12.30)	62.00 (9.466)	615.0 (15.20)	60.00 (9.100)
	Farmer and fisher	384.0 (15.60)	89.00 (13.59)	438.0 (10.80)	78.00 (11.75)
	Blue-collar worker	508.0 (20.60)	107.0 (16.40)	135.0 (3.300)	9.000 (1.355)
	Elementary occupations	209.0 (8.500)	64.00 (9.771)	478.0 (11.80)	67.00 (10.20)
	Unemployed (housewife, etc.)	464.0 (18.80)	234.0 (35.73)	1962 (48.40)	429.0 (65.00)
Household incomes (no. of participants, %)					
	High class	728.0 (29.50)	138.0 (21.07)	1126 (27.78)	106.0 (16.10)
	Middle class	1272 (51.54)	324.0 (49.60)	2035 (50.20)	290.0 (43.90)
	Low class	468.0 (18.96)	191.0 (29.20)	893.0 (22.03)	264.0 (40.00)
Alcohol consumption (No. of participants, %)					
	Frequently drinks	979.0 (39.67)	251.0 (38.32)	313.0 (7.721)	32.00 (4.800)
	Occasionally drinks	584.0 (23.66)	121.0 (18.47)	648.0 (15.98)	55.00 (8.300)
	Rarely drinks	434.0 (17.59)	114.0 (17.41)	1452 (35.82)	192.0 (29.10)
	Never drinks	295.0 (11.95)	115.0 (17.56)	639.0 (15.76)	130.0 (19.70)
	Nonapplicable	176.0 (7.131)	52.00 (8.000)	1002 (24.72)	251.0 (38.10)
Sleep duration (mean, SD)		6.926 (3.474)	6.860 (1.478)	6.744 (3.211)	6.930 (6.440)
Vital signs (mean, SD)					
	Pulse rate per 15 sec	17.31 (2.225)	18.07 (2.668)	17.41 (2.147)	18.36 (2.465)
	Systolic BP (mmHg)^‡^	121.9 (15.99)	127.7 (16.20)	119.30 (17.72)	130.1 (18.14)
	Diastolic BP (mmHg) ^‡^	80.02 (10.62)	79.50 (10.55)	76.13 (10.26)	76.78 (9.783)
Anthropometrics (mean, SD)					
	Body mass index (kg/m^2^)	23.51 (2.935)	24.72 (2.974)	23.44 (3.041)	25.26 (3.411)
	Visceral adiposity index	4.726 (5.734)	6.675 (8.348)	4.764 (3.951)	7.807 (6.049)
	Height (cm) ^‡^	168.3 (6.189)	167.2 (6.272)	155.5 (5.967)	153.6 (5.840)
	Weight (kg) ^‡^	66.76 (10.00)	69.28 (10.08)	56.72 (8.295)	59.68 (9.384)
	Waist circumference (cm) ^‡^	83.42 (8.393)	88.29 (8.810)	78.89 (8.803)	86.59 (9.395)
	Waist-to-height ratio^‡^	0.496 (0.050)	0.528 (0.052)	0.508 (0.060)	0.564 (0.062)
Blood parameters (mean, SD)					
	Total cholesterol (mg/dL) ^‡^	188.7 (34.29)	182.6 (40.67)	193.6 (34.68)	196.2 (41.02)
	HDL cholesterol (mg/dL) ^‡^	45.85 (10.95)	42.48 (9.920)	50.05 (11.02)	45.22 (10.70)
	Triglyceride (mg/dL) ^‡^	150.5 (124.1)	194.8 (186.6)	116.3 (72.67)	165.1 (98.81)
	Ferritin (ng/mL) ^‡^	120.1 (124.7)	164.6 (294.5)	50.63 (43.56)	73.59 (65.27)
	Aspartate aminotransferase (IU/L) ^‡^	24.64 (14.19)	27.71 (18.80)	20.73 (6.986)	23.96 (11.80)
	Alanine aminotransferase (IU/L) ^‡^	24.06 (14.91)	30.21 (23.06)	17.55 (10.12)	23.92 (16.34)
	Alkaline phosphatase (IU/L) ^‡^	233.5 (64.69)	252.0 (92.06)	220.1 (71.56)	258.4 (86.62)
	Hematocrit (%) ^‡^	44.28 (3.208)	43.41 (3.781)	38.95 (2.966)	39.30 (3.135)
	Vitamin D (ng/dL) ^‡^	21.06 (7.233)	20.19 (7.250)	17.82 (6.700)	18.50 (7.471)
	Blood urea nitrogen (mg/dL) ^‡^	15.41 (4.507)	16.53 (4.920)	14.22 (3.978)	15.64 (5.020)
	Creatinine (mg/dL) ^‡^	0.946 (0.162)	0.990 (0.358)	0.708 (0.157)	0.750 (0.242)
Bone mineral density (mean, SD)					
	Ward BMD (g/cm^2^) ^‡^	0.560 (0.134)	0.530 (0.128)	0.523 (0.156)	0.440 (0.145)
	Lumbar spine BMD (g/cm^2^) ^‡^	0.944 (0.142)	0.980 (0.150)	0.881 (0.163)	0.840 (0.149)
	Left rib BMD (g/cm^2^) ^‡^	0.686 (0.086)	0.710 (0.088)	0.619 (0.086)	0.600 (0.079)
	Right rib BMD (g/cm^2^) ^‡^	0.688 (0.078)	0.700 (0.085)	0.623 (0.078)	0.600 (0.076)
	Thoracic spine BMD (g/cm^2^) ^‡^	0.919 (0.139)	0.960 (0.148)	0.808 (0.149)	0.780 (0.133)
	Pelvis BMD (g/cm^2^) ^‡^	1.098 (0.157)	1.120 (0.158)	1.029 (0.161)	0.990 (0.163)
Fat mass (mean, SD)					
	Trunk fat (g) ^‡^	7982 (3149)	9511 (3011)	9699 (3222)	11870 (3482)
	Left leg fat (g) ^‡^	1976 (685.6)	1991 (654.7)	2998 (817.8)	2705 (829.5)
	Right leg fat (g) ^‡^	2016 (702.7)	2046 (671.6)	3069 (842.6)	2781 (853.4)
	Body total fat (g) ^‡^	13479 (4859)	15212 (4588)	18058 (5052)	19912 (5392)
Muscle mass (mean, SD)					
	Trunk muscle (g) ^‡^	24178 (3213)	25169 (3534)	18162 (2303)	19158 (2775)
	Left leg muscle (g) ^‡^	7877 (1231)	7823 (1265)	5435 (810)	5468 (880)
	Right leg muscle (g) ^‡^	8045 (1230)	7968 (1276)	5537 (819)	5563 (903)
	Body total muscle (g) ^‡^	45470 (6166)	46181 (6595)	32284 (4103)	33436 (4786)

The data are represented as numbers of participants and percentages, N (%), or as the mean and standard deviation (SD) for continuous and categorical variables, respectively. ^†^ p<0.05 and ^‡^ p<0.001. These results indicate significant differences between men and women without stratification into groups, as determined using independent two-sample t-tests [means (standard deviations)]. HDL, high-density lipoprotein; BMD, bone mineral density. Frequently drinks: drinking more than twice a week. Occasionally drinks: drinking from twice to four times a month. Rarely drinks: drinking less than or equal to once a month. Sleep duration: the average daily amount of sleep.

## Results

Women with T2DM were older than men with T2DM. Various indices, such as the BMI, VAI, WHtR and body fat mass, were significantly higher in women with T2DM than in men with T2DM. The WC was higher in men than in women, and the BMD was lower in women than in men.

Tables 2 and 3 list the associations of T2DM with anthropometric indices, BMD, body fat and muscle mass in Korean men and women. Among all the variables, the WHtR displayed the strongest association with T2DM in men in the crude analysis (OR = 1.951 [95% CI, 1.770–2.150], p≤0.001), and the association remained highly significant after adjusting for age and BMI (adjusted OR = 1.838 [1.513–2.233], adjusted p≤0.001). Of the BMD variables, the thoracic spine BMD displayed the strongest association with T2DM in both crude (OR = 1.347 [1.238–1.465], p≤0.001) and adjusted analyses (adjusted OR = 1.222 [1.118–1.336], adjusted p≤0.001). Among the body fat variables, trunk fat exhibited the strongest association with T2DM in the crude analysis (OR = 1.612 [1.477–1.760], p≤0.001), and the association remained highly significant after adjusting for age and BMI (adjusted OR = 1.436 [1.237–1.667], adjusted p≤0.001). Of the muscle mass variables, trunk muscle mass displayed the strongest association with T2DM in both the crude (OR = 1.346 [1.235–1.467], p≤0.001) and adjusted analyses (adjusted OR = 1.375 [1.203–1.571], adjusted p≤0.001).

**Table 2:** Associations of diabetes with anthropometrics, BMD, body fat and muscle mass in Korean men.

Variables		Crude		Adjustment		AUCs
		P	OR	P	OR	
Age		<0.001	1.607 (1.472–1.755)	-	-	0.644 (0.622–0.666)
Anthropometrics						
	Body mass index	<0.001	1.502 (1.375–1.641)	-	-	0.616 (0.592–0.640)
	Visceral adiposity index	<0.001	1.358 (1.226–1.503)	<0.001	1.299 (1.172–1.439)	0.626 (0.602–0.650)
	Height	<0.001	0.840 (0.771–0.916)	0.770	1.015 (0.920–1.119)	0.553 (0.528–0.578)
	Weight	<0.001	1.282 (1.177–1.398)	0.771	1.029 (0.849–1.247)	0.575 (0.551–0.600)
	Waist circumference	<0.001	1.804 (1.640–1.983)	<0.001	1.769 (1.472–2.127)	0.662 (0.639–0.685)
	Waist-to-height ratio	<0.001	1.951 (1.770–2.150)	<0.001	1.838 (1.513–2.233)	0.680 (0.658–0.703)
Bone mineral density						
	Ward BMD	<0.001	0.807 (0.738–0.882)	0.315	0.945 (0.847–1.055)	0.563 (0.538–0.587)
	Lumbar spine BMD	<0.001	1.251 (1.148–1.364)	<0.001	1.188 (1.083–1.304)	0.560 (0.535–0.585)
	Left rib BMD	<0.001	1.237 (1.135–1.349)	0.002	1.156 (1.054–1.269)	0.568 (0.543–0.593)
	Right rib BMD	<0.001	1.215 (1.115–1.323)	0.007	1.140 (1.037–1.252)	0.554 (0.528–0.579)
	Thoracic spine BMD	<0.001	1.347 (1.238–1.465)	<0.001	1.222 (1.118–1.336)	0.589 (0.564–0.613)
	Pelvis BMD	0.001	1.156 (1.062–1.258)	0.012	1.137 (1.029–1.256)	0.549 (0.524–0.573)
Fat mass						
	Trunk fat	<0.001	1.612 (1.477–1.760)	<0.001	1.436 (1.237–1.667)	0.643 (0.620–0.665)
	Left leg fat	0.600	1.023 (0.939–1.115)	<0.001	0.639 (0.563–0.724)	0.506 (0.482–0.531)
	Right leg fat	0.317	1.045 (0.959–1.138)	<0.001	0.657 (0.579–0.745)	0.513 (0.489–0.538)
	Body total fat	<0.001	1.421 (1.304–1.549)	0.520	1.049 (0.907–1.214)	0.608 (0.584–0.631)
Muscle mass						
	Trunk muscle	<0.001	1.346 (1.235–1.467)	<0.001	1.375 (1.203–1.571)	0.583 (0.558–0.608)
	Left leg muscle	0.325	0.957 (0.878–1.044)	<0.001	0.796 (0.703–0.902)	0.511 (0.485–0.536)
	Right leg muscle	0.160	0.940 (0.862–1.025)	<0.001	0.773 (0.682–0.876)	0.514 (0.488–0.539)
	Body total muscle	0.010	1.119 (1.027–1.220)	0.977	1.002 (0.876–1.146)	0.533 (0.508–0.559)

The results were obtained from the crude analysis and analyses adjusted for age and BMI using binary logistic regression analyses. AUC values were calculated by creating ROC curves using SPSS software. OR, odds ratio; AUC, area under the receiver operating characteristic curve.

**Table 3:** Associations of diabetes with anthropometrics, BMD, body fat and muscle mass in Korean women.

Variables		Crude		Adjustment		AUCs
		P	OR	P	OR	
Age		<0.001	2.134 (1.958–2.327)	-	-	0.720 (0.701–0.740)
Anthropometrics						
	Body mass index	<0.001	1.715 (1.583–1.858)	-	-	0.661 (0.638–0.683)
	Visceral adiposity index	<0.001	1.686 (1.567–1.814)	<0.001	1.415 (1.313–1.524)	0.708 (0.687–0.728)
	Height	<0.001	0.728 (0.670–0.790)	0.070	1.095 (0.993–1.207)	0.591 (0.568–0.613)
	Weight	<0.001	1.389 (1.284–1.503)	0.068	1.182 (0.987–1.414)	0.596 (0.572–0.620)
	Waist circumference	<0.001	2.305 (2.109–2.519)	<0.001	2.465 (2.074–2.930)	0.730 (0.709–0.750)
	Waist-to-height ratio	<0.001	2.494 (2.276–2.733)	<0.001	2.390 (1.990–2.870)	0.747 (0.728–0.766)
Bone mineral density						
	Ward BMD	<0.001	0.545 (0.497–0.599)	0.022	0.855 (0.748–0.970)	0.663 (0.640–0.685)
	Lumbar spine BMD	<0.001	0.767 (0.706–0.835)	0.009	1.164 (1.038–1.304)	0.581 (0.559–0.604)
	Left rib BMD	<0.001	0.794 (0.724–0.871)	0.744	1.019 (0.911–1.139)	0.567 (0.543–0.591)
	Right rib BMD	<0.001	0.777 (0.713–0.847)	0.612	1.029 (0.922–1.148)	0.579 (0.555–0.602)
	Thoracic spine BMD	<0.001	0.806 (0.738–0.881)	0.148	1.073 (0.975–1.180)	0.560 (0.537–0.583)
	Pelvis BMD	<0.001	0.751 (0.688–0.820)	0.497	1.037 (0.933–1.154)	0.586 (0.563–0.610)
Fat mass						
	Trunk fat	<0.001	1.856 (1.710–2.015)	<0.001	1.741 (1.480–2.047)	0.682 (0.660–0.703)
	Left leg fat	<0.001	0.675 (0.616–0.740)	<0.001	0.367 (0.321–0.419)	0.606 (0.582–0.630)
	Right leg fat	<0.001	0.689 (0.629–0.754)	<0.001	0.375 (0.329–0.428)	0.601 (0.576–0.625)
	Body total fat	<0.001	1.407 (1.300–1.523)	0.005	0.793 (0.674–0.934)	0.603 (0.579–0.626)
Muscle mass						
	Trunk muscle	<0.001	1.483 (1.370–1.606)	<0.001	1.628 (1.447–1.831)	0.605 (0.581–0.630)
	Left leg muscle	0.331	1.041 (0.960–1.130)	0.471	1.041 (0.933–1.161)	0.513 (0.489–0.537)
	Right leg muscle	0.394	1.036 (0.955–1.125)	0.713	1.021 (0.914–1.140)	0.509 (0.485–0.534)
	Body total muscle	<0.001	1.300 (1.200–1.408)	<0.001	1.345 (1.199–1.510)	0.573 (0.548–0.598)

The results were obtained from the crude analysis and analyses adjusted for age and BMI using binary logistic regression analyses. AUC values were calculated by creating ROC curves using SPSS software. OR, odds ratio; AUC, area under the receiver operating characteristic curve.

In women, the WHtR displayed the strongest association with T2DM in the crude analysis among all the variables (OR = 2.494 [2.276–2.733], p≤0.001), and the association remained highly significant after adjusting for age and BMI (adjusted OR = 2.390 [1.513–2.233], adjusted p≤0.001). Of the BMD variables, ward BMD was negatively associated with T2DM in both the crude (OR = 0.545 [0.497–0.599], p≤0.001) and adjusted analyses (adjusted OR = 0.855 [0.748–0.970], adjusted p = 0.022). Among the fat mass variables, the trunk fat mass displayed the strongest association with T2DM in the crude analysis (OR = 1.856 [1.710–2.015], p≤0.001), and the association remained highly significant after adjusting for age and BMI (adjusted OR = 1.741 [1.480–2.047], adjusted p≤0.001). Of the muscle mass variables, trunk muscle mass displayed the strongest association with T2DM in both crude (OR = 1.483 [1.370–1.606], p≤0.001) and adjusted analyses (adjusted OR = 1.628 [1.447–1.831], adjusted p≤0.001).

In the comparison of the predictive power of all variables for identifying T2DM based on the AUC, the WHtR exhibited the highest AUC value in both men (AUC = 0.680 [0.658–0.703]) and women (AUC = 0.747 [0.728–0.766]).

## Discussion

T2DM is a representative metabolic disease associated with various diseases, such as hypertension [49,50], heart disease, stroke [51–53], kidney disease [54] and fracture [55–57]. In the present study, we identified the associations and the best indicator of T2DM among anthropometrics, BMD and body composition variables in Korean adults.

Our findings indicate that WC and WHtR are the best indicators of T2DM in Korean adults and showed that the power of general anthropometric indices for the identification of T2DM was better than those of BMD and body composition variable. The WC and WHtR are known as obesity indicators. Numerous studies have investigated the association of obesity indicators with T2DM and attempted to identify the best indicators of T2DM. As shown in the study by Tulloch-Reid and colleagues [9], BMI and WHtR are excellent predictors of T2DM risk in Pima Indians. Cabrera and colleagues [10] and Xu and colleagues [11] reported that WHtR was the main predictor of diabetes in approximately 5521 adults in the Canary Islands and was the best predictor for T2DM in approximately 7,567 Chinese adults aged 20–79 years. In recent studies by Zhang and colleagues and Mirzaei and colleagues [12,13], WHtR was also the best indicator of diabetes in approximately 1,185 adults aged 40–89 years in Southwest China and approximately 9,293 adults of Yazd City in Iran. According to Kodama and colleagues [8], Browning and colleagues [14], and Rådholm and colleagues [15] from the ADVANCE-ON study, WHtR might be an effective screening tool for diabetes. As another indicator of diabetes, WC was significantly associated with T2DM in the Henan Rural Cohort Study [16], in a cross-sectional study [17], and in 11,937 Korean adults [18]. The CVAI was strongly associated with T2DM in approximately 2,383 Chinese adults in a study by Wu and colleagues [20], and age had the greatest impact on diabetes in approximately 5,573 participants in Ghana, according to the study by Nawfal and colleagues [21]. Because body fat distribution is associated with T2DM [25,26], Vasan and colleagues showed that leg fat is strongly associated with T2DM [27]. Our finding was consistent with the results of previous studies [8–15], indicating that WC and WHtR had the strongest predictive power for T2DM.

In addition, diabetes affects bone metabolism [30–32]. We analyzed the association of T2DM with BMD, and our findings showed that T2DM was associated with the thoracic spine BMD in men, and ward BMD in women. Many studies have also shown that T2DM is associated with higher BMD and, paradoxically, with increased fracture risk [28–31]. According to Strotmeyer and colleagues [32], in 2,979 white and black men and women aged 70–79 years, T2DM was associated with higher hip BMD, and lower spine bone volume. Dennison and colleagues [33] reported that the total femur and femoral neck BMD had a positive correlation with IR, with stronger results observed in women. As shown in the study by Vestergaard P [34], the BMD Z-score is increased in the spine and hip in patients with T2DM and the increase in fracture risk is higher and BMD is lower in participants with complications from diabetes. According to an analysis based on 15 observational studies of approximately 3,437 participants with diabetes and 19,139 control subjects by Ma and colleagues [36], significantly higher BMD of the femoral neck, hip and spine is observed in patients with T2DM. The BMD in the left femur and total hip was significantly greater in insulin-resistant women in a study by Arikan and colleagues [37]. In contrast, several studies have shown that diabetes affects bone loss [33,38]. According to an analysis of a 4‐year change in BMD data from a cohort of white and black well‐functioning men and women 70‐79 years of age in a study by Dennison [33], white women with diabetes had more rapid bone loss at the femoral neck than women with a normal glucose metabolism. As shown by changes in the BMD data of 4,960 Canada women aged ≥40 years over a period of approximately 4.3 years in the study of Leslie and colleagues [38], diabetes was associated with a slightly greater BMD loss at the femoral neck but not at other measurement sites. In addition, participants with both T2DM and fracture have a low BMD [39,40]. In 150 older women with T2DM observed by Yamamoto and colleagues in Japan [39], prevalent vertebral fractures were associated with a lower, but not significantly different, lumbar spine BMD Z-score. In a study of 150 older women with T2DM in England by Patel and colleagues [40], participants with a previous fracture had lower lumbar-spine and total-hip BMD Z scores, but the differences were not statistically significant. As shown in a report analyzing data from the MrOS study [41], which enrolled 5,994 men aged ≥65 years from March 2000 through April 2002 [42], T2DM is not associated with a higher prevalence or incidence of vertebral fractures in older men, and a higher spine BMD is associated with a lower prevalence and incidence of vertebral fractures in patients with T2DM. In men, our finding was consistent with the results of previous studies [34–37], indicating that the BMD was increased in various sites such as the hip, lumbar spine, and thoracic spine. However, in women, our finding contrasted with that of Arikan and colleagues [37], indicating that the BMD in the left femur and total hip was significantly greater in insulin-resistant women. Through the previous studies, we know that T2DM influences bone metabolism and fracture, but the correlation of T2DM with BMD remains inconsistent across studies.

Some studies have investigated gender differences in correlations between T2DM and obesity measures [8], such as BMI, WC, waist-to-hip ratio (WHR) and WHtR, as well as BMD. Our findings showed that various indices such as age, BMI, VAI, WHtR and body fat mass in patients with T2DM were significantly higher in women than in men, and the increase in WC was also higher in women than in men; however, the BMD was lower in women than in men, probably because of the older age of the women. According to the Henan Rural Cohort study of 38,466 participants aged 18–79 years conducted by Tian and colleagues [16], there are gender-specific differences between obesity measures and T2DM. In women, WC is independently associated with an increased risk of T2DM, regardless of BMI status, whereas both BMI and WC positively correlate with the risk of T2DM in men. Sallam and colleagues investigated the gender differences among participants with T2DM (64 men and 50 women) with and without cardiovascular disease (CVD) [17]. Age, body weight, BMI, and systolic blood pressure (SBP) were significantly higher in women with CVD than in participants without CVD. Rianon and colleagues reported that BMD was not altered for women with T2DM but was significantly higher in men with T2DM compared with men without diabetes, in approximately 149 Mexican American older men and women [41]. Our results support the findings from recent studies identifying gender differences in correlations of T2DM with WC, BMI, and BMD [16,17,41].

The present study had several limitations. First, cause-effect associations were difficult to determine because of the cross-sectional design. Second, our results were limited to Korean adults because we used data from the KNHANES in this study. Third, since KNHANES does not contain detailed information on medication use, a more precise analysis was not possible. Fourth, a large number of values were missing in the original data samples, resulting in the exclusion of a number of the samples during the sample selection process, and only 36.78% of samples were selected. Therefore, we were unable to conclude that the results of this paper represent the frequency of T2DM in Koreans. Fifth, the datasets from KNHANES may be affected by response bias because self-reported data from participants such as "the current use of antidiabetic medications or glucose-lowering drugs, or physician-diagnosed diabetes" are also used in the diagnosis of T2DM.

## Conclusions

In the present study, we evaluated the associations of T2DM with anthropometrics, BMD, body fat mass and muscle mass in Korean adults. The WHtR in men and LL and RL fat in women displayed the strongest associations with T2DM, and the WC and WHtR in both men and women exhibited the highest predictive power for T2DM. Moreover, age, VAI, WC and WHtR exhibited greater predictive power in women than in men. Our findings provide clinical information that may identify patients with T2DM during initial screening steps. Further studies are needed to build a model for accurate identification based on a combination of BMD, anthropometric, and fat mass data.
